# Construction of a predictive model for postoperative hospitalization time in colorectal cancer patients based on interpretable machine learning algorithm: a prospective preliminary study

**DOI:** 10.3389/fonc.2024.1384931

**Published:** 2024-06-14

**Authors:** Zhongjian Wen, Yiren Wang, Shouying Chen, Yunfei Li, Hairui Deng, Haowen Pang, Shengmin Guo, Ping Zhou, Shiqin Zhu

**Affiliations:** ^1^ School of Nursing, Southwest Medical University, Luzhou, China; ^2^ Wound Healing Basic Research and Clinical Application Key Laboratory of Luzhou, Southwest Medical University, Luzhou, China; ^3^ Department of Oncology, The Affiliated Hospital of Southwest Medical University, Luzhou, China; ^4^ Department of Nursing, The Affiliated Hospital of Southwest Medical University, Luzhou, China; ^5^ Department of Radiology, The Affiliated Hospital of Southwest Medical University, Luzhou, China; ^6^ Department of Endocrinology and Metabolism, The Affiliated Hospital of Southwest Medical University, Luzhou, China

**Keywords:** machine learning, predictive model, colorectal cancer, prospective study, explainable algorithm, hospital stay

## Abstract

**Objective:**

This study aims to construct a predictive model based on machine learning algorithms to assess the risk of prolonged hospital stays post-surgery for colorectal cancer patients and to analyze preoperative and postoperative factors associated with extended hospitalization.

**Methods:**

We prospectively collected clinical data from 83 colorectal cancer patients. The study included 40 variables (comprising 39 predictor variables and 1 target variable). Important variables were identified through variable selection via the Lasso regression algorithm, and predictive models were constructed using ten machine learning models, including Logistic Regression, Decision Tree, Random Forest, Support Vector Machine, Light Gradient Boosting Machine, KNN, and Extreme Gradient Boosting, Categorical Boosting, Artificial Neural Network and Deep Forest. The model performance was evaluated using Bootstrap ROC curves and calibration curves, with the optimal model selected and further interpreted using the SHAP explainability algorithm.

**Results:**

Ten significantly correlated important variables were identified through Lasso regression, validated by 1000 Bootstrap resamplings, and represented through Bootstrap ROC curves. The Logistic Regression model achieved the highest AUC (AUC=0.99, 95% CI=0.97–0.99). The explainable machine learning algorithm revealed that the distance walked on the third day post-surgery was the most important variable for the LR model.

**Conclusion:**

This study successfully constructed a model predicting postoperative hospital stay duration using patients’ clinical data. This model promises to provide healthcare professionals with a more precise prediction tool in clinical practice, offering a basis for personalized nursing interventions, thereby improving patient prognosis and quality of life and enhancing the efficiency of medical resource utilization.

## Graphical Abstract

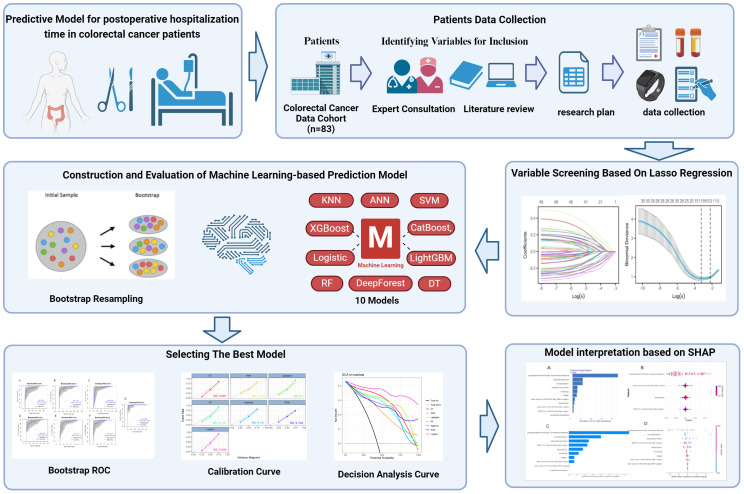

## 1 Introduction

Colorectal cancer (CRC) is one of the most common malignancies, with both its incidence and mortality rates on an upward trend, showing a notable shift toward younger patients (1). With surgical treatment becoming the primary method for CRC management, the economic burden of CRC treatment remains high (2). To alleviate the financial strain on patients and their families and to enhance hospital resource utilization, more efficient management plans are needed.

Length of stay (LOS) objectively reflects a patient’s recovery of physical function and serves as an indicator of healthcare efficiency. Postoperative hospital stays are often characterized by prolonged bed rest and sedation, where an extended LOS (pLOS) not only increases the economic and psychological burden on patients and their families but also raises the risk of complications and hospital-acquired infections (3). To mitigate the strain on healthcare resources and alleviate the social and psychological pressures on patients, it is crucial to establish predictive models that identify patients at risk of pLOS during their treatment and to promptly recognize risk factors for timely nursing interventions, thereby accelerating patients’ recovery and reducing hospital stay durations.

Machine learning demonstrates multiple advantages in binary prediction models, offering high predictive accuracy, automatic key feature selection and extraction, and capturing nonlinear relationships between features (4, 5). Existing predictive models for the hospital stay duration of CRC patients are retrospective, limiting their predictive accuracy for future trends. Previous study has developed a Support Vector Machine (SVM) model to differentiate the risk of extended postoperative hospital stays in CRC patients, with the SVM model showing an AUC of 0.821 in the validation set, demonstrating the potential of machine learning-based models in binary classification problems (3). However, current models face two main issues: a lack of interpretability, making model results difficult to explain, and a small sample size, limiting the training dataset and affecting model generalizability and accuracy (6–8). Bootstrap resampling can better utilize limited data to provide a more robust assessment of model performance. It involves random sampling with replacement from the training dataset to create multiple subsets for model validation, reducing the variance of validation results and ensuring more reliable evaluations compared to proportional splits, especially with small sample sizes (9, 10). SHAP (SHapley Additive exPlanations), based on cooperative game theory, offers clear explanations for feature contribution values, bridging the gap between complex algorithms and clinical application, ensuring transparency and traceability in model-based decision-making, which is crucial for the scientific validity and credibility of medical decisions (11, 12).

This study aims to develop a predictive model for the hospital stay duration of CRC patients using prospective data and explainable machine learning algorithms, employing Bootstrap resampling for robust model performance. The model predicts the risk of pLOS and identifies perioperative factors potentially influencing hospital stay duration.

## 2 Methods

### 2.1 Patient recruitment

This study was conducted in accordance with the Declaration of Helsinki (revised in 2013) and received approval from the Ethics Review Committee of the Affiliated Hospital of Southwest Medical University (No.20190321–12). All participants signed informed consent forms. Patients with colorectal cancer admitted to the gastrointestinal surgery ward of the Affiliated Hospital of Southwest Medical University from January to October 2019 were selected for this study. Inclusion criteria were: (1) pathologically diagnosed with colorectal cancer and underwent laparoscopic colorectal cancer radical surgery; (2) aged 18 to 70 years; (3) Preoperative ability to walk without mobility limitations, with muscle strength > Grade 3; (4) no severe preoperative cardiac, pulmonary, or renal dysfunction; (5) patient informed consent and voluntary participation in the study. Exclusion criteria included: (1) history of psychiatric disorders and cognitive impairments, unable to complete questionnaire assessments; (2) diseases prohibiting movement; (3) palliative surgery or neoadjuvant chemoradiotherapy. Drop-out criteria were: (1) conversion from laparoscopic to open surgery; (2) postoperative ICU transfer or transfer to another department; (3) postoperative hospital stay<3 days; (4) severe postoperative cardiac, pulmonary, or renal diseases; (5) non-compliant wearing of the wireless smart pedometer; (6) withdrawal from the study for various reasons.

### 2.2 Variable selection and definition of target variables

Prospective clinical data were collected based on previous literature and expert consultations. Data included demographic and social characteristics (age, gender, occupation, education level, health insurance status), lifestyle history (smoking and drinking history), laboratory tests (preoperative albumin and hemoglobin levels), past medical and surgical history (surgery duration, intraoperative blood loss), disease status (tumor location, clinical stage, underlying diseases), preoperative Barthel score, Karnofsky Performance Status (KPS), Zubrod Performance Status (ZPS), postoperative day three patient mobility data (steps, time, distance) recorded by wireless smart pedometers, preoperative and postoperative day three pain scores, 15-item Quality of Recovery (QOR-15) scores, and complications (intestinal obstruction, urinary tract infections, anastomotic fistula, urinary retention, pulmonary infections) (13–15).

Prolonged LOS (pLOS) was defined as greater than the average or median value (16, 17). Due to the variability in patient care, management, and treatment responses, the median, as a measure of central tendency, was deemed more appropriate for classifying extended hospital stays than the mean. LOS was defined as the interval from the day of surgery to the day of discharge. According to existing studies, the median postoperative hospital stay for colorectal cancer patients is 8 days, thus patients with a LOS of 8 days or less were classified into the ideal LOS (iLOS) group, and those with a stay of more than 9 days were defined as the pLOS group (3).

### 2.3 Variable screening by Lasso regression

Lasso regression, a regularized linear regression method widely applied for variable selection, performs variable selection and complexity adjustment while fitting generalized linear models. In this study, Lasso regression was used for variable screening. The regression controls the number of selected variables by adjusting the λ parameter, where a larger λ implies a greater penalty and fewer retained variable features. Optimal penalty parameters were chosen through cross-validation, selecting the λ value with the smallest error to identify the most relevant variables. The Lasso regression formula is as follows:


$$\underset{\beta }{\text{min}}\{\frac{1}{2n}||y+X{\beta ||}_{2}^{2}+\lambda ||{\beta ||}_{1}\}$$

In the formula, y represents the vector of response variables. X is the design matrix that contains observations of p explanatory variables across n samples. β is the vector of regression coefficients, representing the impact of each explanatory variable on the response variable. *λ* is the regularization parameter that controls the strength of the penalty term.

Selected variables were then used as candidate important variables for further model construction. Statistical analysis and visualization were conducted in R version 4.2.1, using the glmnet package to analyze the cleaned data for variable lambda values, likelihood values, and data visualization.

### 2.4 Construction of machine learning models

To confirm the discriminatory power of clinical features related to hospital stay duration, ten machine learning algorithms were utilized, including Logistic Regression, Decision Tree (DT), Random Forest (RF), Support Vector Machine (SVM), Light Gradient Boosting Machine (lightGBM), KNN, Extreme Gradient Boosting (XGBoost), Categorical Boosting (CatBoost), Artificial Neural Network (ANN) and Deep Forest. Models were trained using cross-validation with grid search to automatically find optimal hyperparameters for best model performance (**Supplementary Files 1**). Given the study’s data characteristics, Bootstrap resampling was used for internal validation to ensure model robustness and reliability. This method involves random sampling with replacement from the original dataset to generate multiple Bootstrap samples. Classification models were trained on each Bootstrap sample, and their ROC curve AUCs were calculated. Bootstrap ROC curves were drawn based on all Bootstrap samples to evaluate model predictive performance and provide a comprehensive assessment. Bootstrap resampling for internal validation can better utilize limited data to provide a more robust assessment of model performance, reducing the variance of validation results and ensuring more reliable evaluations compared to proportional splits, especially with small sample sizes.

Model calibration was assessed using calibration curves, comparing predicted event probabilities with actual event frequencies. This study also used Decision Curve Analysis (DCA) for clinical net benefit analysis. DCA offers a method to evaluate the predictive performance of classification models in the medical field by assessing the clinical net benefit of all models, aiding healthcare decision-makers in choosing the most appropriate model for specific clinical contexts. The X-axis represents the patient threshold, and the Y-axis represents the net benefit. Each model’s curve shows the net benefit compared to the baseline decision (such as all treatment or no treatment at all) at various thresholds (18).

### 2.5 Model interpretation based on SHAP

To analyze the contribution of variables to the prediction of hospital stay duration for colorectal cancer patients, the SHAP (Shapley Additive exPlanations) algorithm was employed using the DALEX and fastshap packages for model interpretation. Initial steps included calculating global mean absolute SHAP values to determine the overall importance of model features. Further, the impact of each variable’s SHAP values on the model’s predictive outcomes was explored.

### 2.6 Statistical analysis

Statistical analyses were performed using the ‘stats’ package in R (version 4.2.1), selecting appropriate statistical methods based on data characteristics. Quantitative data were described using M (P25, P75) following normality and homogeneity of variance tests, with independent sample t-tests or rank sum tests for between-group comparisons and repeated measures ANOVA for different time points. Categorical data were presented as frequencies and proportions, with χ2 tests or Fisher’s exact tests for between-group comparisons, and rank sum tests for ordinal data. A two-sided p-value<0.05 was considered statistically significant. The area under the curve (AUC) was used to evaluate the performance of the constructed models.

## 3 Results

### 3.1 Baseline information analysis

Statistical analysis of 84 patients showed that the median length of stay (LOS) was 8 days, with 47 patients (56.63%) having a LOS of ≤8 days. The average age of patients was 58 years, with a gender distribution of 47 males to 36 females. Preoperative variables included age, gender, smoking status, tumor stage, and location, among 19 variables in total, while postoperative variables included surgery duration, blood loss, mobility data for the first three days post-surgery, QOR-15, and pain scores, among 20 variables in total (**Supplementary Files 2**). Statistically significant preoperative features included smoking (P=0.006) and education level (P=0.150), while perioperative features with statistical significance included intraoperative blood loss (P=0.004), steps count on the first day after surgery (P<0.01), second day (P<0.01), and third day (P<0.01), postoperative first-day movement distance (P<0.01), second day (P<0.01), and third day (P<0.01), QOR-15 scores on the first day (P<0.01), second day (P<0.01), and third day (P<0.01), pain scores on the second day after surgery (P=0.022) and third day (P<0.01), and complications (P=0.015).

### 3.2 Feature selection

**Figure 1:**
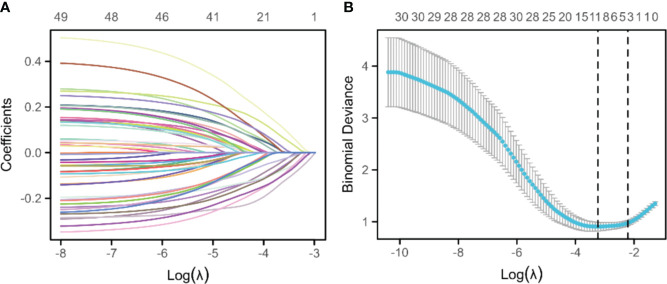
Lasso regression for variable selection. **(A)** The Lasso variable trajectory plot places the logarithm of lambda (log(λ)) on the horizontal axis and the coefficient values of the variables on the vertical axis, revealing the trend of variable coefficients converging toward zero as the lambda parameter increases, thus highlighting the importance of each variable. **(B)** The Lasso coefficient selection plot, with the logarithm of lambda (log(λ)) on the lower x-axis and the count of variables (those with non-zero coefficients at the corresponding lambda value) on the upper x-axis, along with the binomial deviation on the y-axis, demonstrates the variable selection process and the relationship between model deviation and different lambda values.

Through 10-fold cross-validation, the lambda value corresponding to the smallest mean error was found to be lambda. min=0.039433 (standard error=0.08373). This process selected 11 important variables (smoking history, education level, clinical stage, intraoperative blood loss, steps walked on the first day post-surgery, pain scores on the fourth day, distance walked on the third day, QOR-15 scores on the third day) and their corresponding non-zero coefficients (**Figure 1**) for model construction.

### 3.3 Construction and evaluation of machine learning-based predictive models

**Figure 2:**
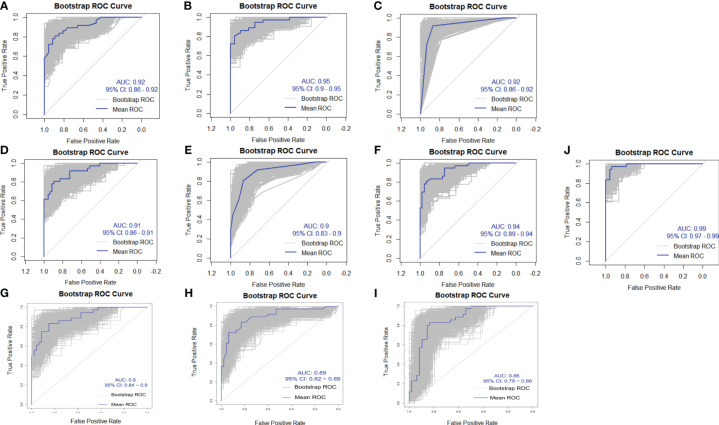
Area Under the ROC Curve (AUC) for ten machine learning models. The horizontal axis (X-axis) represents the false positive rate, while the vertical axis (Y-axis) represents the true positive rate. The models include: **(A)** XGBoost, **(B)** LightGBM, **(C)** DT, **(D)** SVM, **(E)** KNN, **(F)** RF, **(G)** ANN, **(H)** DeepForest, **(I)** CatBoost, and **(J)** Logistic Regression.

**Figure 3:**
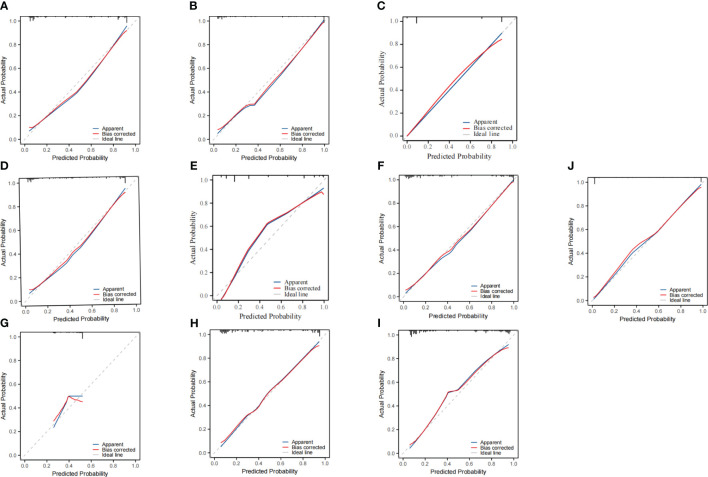
Calibration curves for Ten predictive models. The horizontal axis represents the predicted probability of occurrence by the models, while the vertical axis represents the observed probability of occurrence. The models include: **(A)** XGBoost, **(B)** LightGBM, **(C)** DT, **(D)** SVM, **(E)** KNN, **(F)** RF, **(G)** ANN, **(H)** DeepForest, **(I)** CatBoost, and **(J)** Logistic Regression.

**Figure 4:**
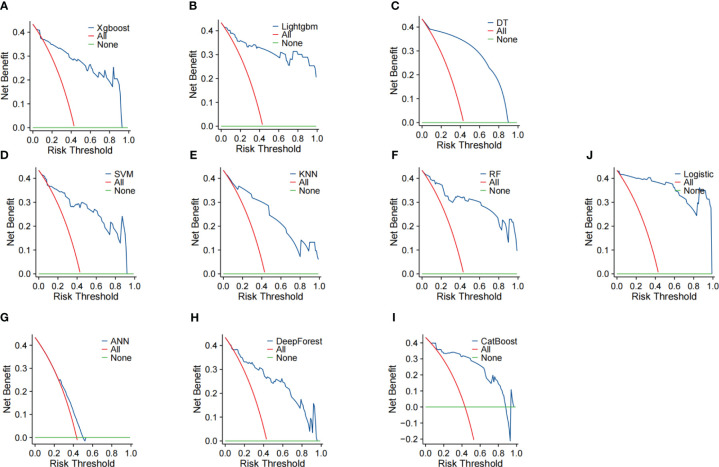
Decision analysis curve of 7 prediction models. The horizontal axis represents the risk probability threshold, and the vertical axis represents the net benefit rate. The models include: **(A)** XGBoost, **(B)** LightGBM, **(C)** DT, **(D)** SVM, **(E)** KNN, **(F)** RF, **(G)** ANN, **(H)** DeepForest, **(I)** CatBoost, and **(J)** Logistic Regression.

Internal validation of the ten models was performed using 1000 bootstrap resamplings. The bootstrap ROC curves of the ten models are shown in **Figure 2**, with the logistic regression model performing the best (AUC=0.99, 95%CI:0.97–0.99), followed by the Lightgbm model (AUC=0.92, 95%CI:0.90–0.95). Calibration curves were used to assess the discrepancy between predicted and actual probabilities, showing good calibration for all models. The logistic regression model demonstrates superior predictive accuracy (**Figure 3**). Decision curve analysis showed that logistic regression had a significant net clinical benefit over the other models across a 0%-100% clinical threshold range, making it the final predictive model based on AUC and clinical utility (**Figure 4**).

### 3.4 Interpretability analysis of the predictive model

The logistic regression-based model for predicting prolonged LOS in postoperative colorectal cancer patients was selected as the final model. The SHAP algorithm was used for interpretability analysis. The logistic regression model, being a linear model derivative from regression to classification, provides a linear decision boundary, making the decision-making process straightforward and interpretable.

Doctors can gain a clearer understanding of predictive models from SHAP plots by visually interpreting the contributions of each feature to the model’s predictions. SHAP plots illustrate the impact of individual features on model output, allowing doctors to identify which factors are impacting the predictions and how they impacting the decision-making process.

**Figure 5:**
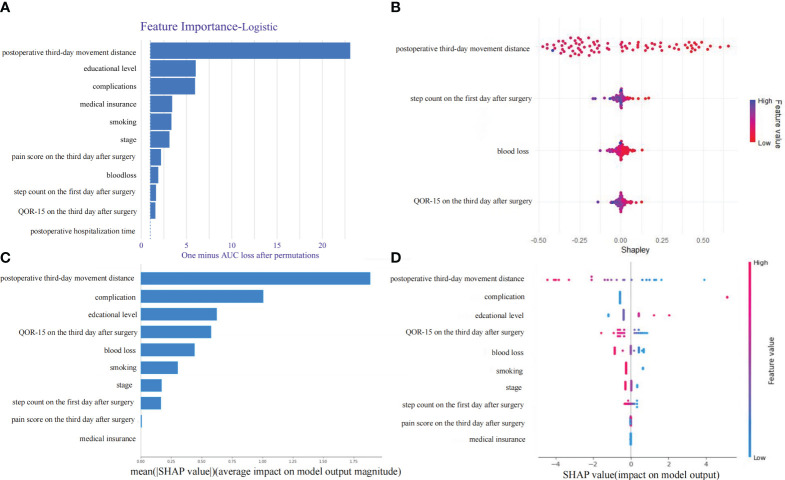
Important variables contribution to the LR (Logistic Regression) model. **(A)** Global variable importance analysis using the DALEX package. **(B)** Continuous variable analysis based on SHAP values. **(C)** The average impact of variables on the magnitude of model output. **(D)** The impact of different variable values on the model output.

Using the DALEX package, the impact of each variable on the prediction was calculated by sequentially removing each feature. The distance walked on the third day post-surgery was found to have the most significant impact on the model’s predictions. Other significant factors included education level, complications, insurance status, smoking history, disease staging, pain scores on the third day, intraoperative blood loss, steps walked on the first day, and QOR-15 scores on the third day (**Figure 5A**).

Global average impacts of each important variable on model output magnitude calculated using the fastshap package showed that the distance walked on the third day post-surgery remained the most significant factor, followed by complications, education level, QOR-15 on the third day, intraoperative blood loss, smoking history, disease stage, steps walked on the first day, and pain scores on the third day (**Figure 5C**). This indicates that the distance walked on the third day post-surgery is the most crucial factor affecting the model’s predictions, making it the primary factor influencing the risk of prolonged LOS for colorectal cancer patients.

**Figure 5B** displays the SHAP value distribution for each feature, with colors indicating the magnitude of feature values—red representing low values and blue representing high values. **Figure 5D** shows the SHAP value distribution for each feature, with the horizontal axis representing SHAP values and the vertical axis representing the features. A SHAP value>0 is associated with an increased risk of extended hospital stay, while a SHAP value<0 is associated with a decreased risk of extended hospital stay. From these figures, we can deduce that the postoperative third-day movement distance is the most significant feature. High values of this feature negatively impact the model output, while low values positively impact the model output. Additionally, the presence of complications and high blood loss significantly negatively affect the model output.

## 4 Discussion

In this study, we constructed ten machine learning models to predict the probability of prolonged length of stay (pLOS) in patients. The optimal model was determined to be the Logistic Regression (LR) model, with an AUC of 0.99 and a 95% CI of 0.97–0.99, demonstrating superior predictive performance. The study also analyzed factors influencing the length of stay (LOS) after surgery for colorectal cancer patients, finding that postoperative mobility had the most significant impact on outcomes. Specific nursing interventions during the perioperative period can help promote patient recovery, reduce hospital stay, and improve hospital resource utilization while reducing patient burden.

Previous studies identified age, gender, marital status, body mass index (BMI), and postoperative complications as influencing factors (6, 17, 19). Variables during surgery such as surgery duration, blood loss, and surgery location were also potential predictors (20). However, previous studies lacked consideration of perioperative factors influencing outcomes. Activity within 24 hours post-surgery was an independent predictor for reducing LOS, and activity on days 1 to 3 post-surgery was crucial for the success of Enhanced Recovery After Surgery (ERAS) programs, reducing moderate to severe postoperative complications.

This study prospectively collected detailed preoperative data and recorded detailed postoperative data for three days, including mobility data (steps, time, distance), sleep duration, pain scores, and the 15-item Quality of Recovery (QOR-15). QOR-15 is a tool for quantifying postoperative walking ability and is an important indicator for assessing physical function recovery from the patient’s perspective (21).

Previous studies have demonstrated the accuracy of machine learning algorithms in predicting pLOS for colorectal cancer surgery patients, improving the reproducibility and generalizability of the developed models (3, 19). Stoean et al. (22) analyzed 368 patients, predicting LOS using SVM, LR, DT, and neural networks, achieving an accuracy of 73.14 ± 4.37 with an ensemble method. Francis et al. (23) included 275 colorectal cancer patients, with a median LOS of 6 days, and constructed a model using MLPNN with an AUC of 0.817, compared to an AUC of 0.807 from logistic regression analysis. The LR model constructed in this study had higher predictive ability and stronger interpretability.

In this study, patient education level and smoking history were significant factors affecting pLOS, which differs from other studies where gender was a significant variable. The median LOS for male patients was 9 days, compared to 7 days for female patients, indicating a lower probability of prolonged pLOS for female patients.

Some studies have shown a significant correlation between patient age and pLOS. However, after feature selection using Lasso, age showed no significant association with prolonged LOS in colorectal cancer patient post-surgery (23, 24). The study divided patients into age groups: ≤62 years (42 cases) and >62 years (41 cases), with no significant difference in LOS between these groups, consistent with Leung et al.’s findings that patient age does not significantly affect hospital stay duration.

Postoperative complications, a relatively constant risk factor affecting LOS, ranked second in variable importance, indicating a strong determinant of LOS (6, 17, 19). Previous research found that male colorectal cancer patients had a higher incidence of postoperative complications than females (25). In this study, males accounted for 75% of the 12 patients with complications. The consistency of these results with previous studies validates the data and modeling methods used in this study. All patients in this study experienced only one complication, with no significant association between preoperative underlying diseases and complications. Postoperative complications primarily included intestinal obstruction, urinary tract infections, anastomotic fistula, urinary retention, and pulmonary infections, with pulmonary and urinary tract infections being the most common in this study’s data. Previous research suggested that preoperative or perioperative factors increase the risk of postoperative complications, emphasizing the need for healthcare professionals to closely monitor patients’ postoperative physical condition to identify and control potential risk factors for complications, further reducing the impact of this variable on LOS and improving postoperative quality of life for patients (26).

While previous research focused on the impact of preoperative physical function levels on LOS, this study emphasized perioperative factors affecting outcomes for colorectal cancer patients (3, 27). The significant positive correlation between postoperative mobility and LOS aligns with the ERAS concept, where scientifically sound early postoperative activity promotes functional recovery (28), reduces complications, and shortens hospital stay (29). Therefore, healthcare professionals developing and assisting patients with early postoperative activity plans can significantly reduce the risk of pLOS, shorten hospital stay, and improve patient satisfaction and healthcare resource utilization.

This study has limitations, including its single-center design and small sample size, which may affect external validity. Bootstrap resampling was used to minimize model overfitting. The study not only considered preoperative variables but also focused on perioperative characteristics to identify more significantly related features for model construction, resulting in a model with excellent predictive performance. The use of interpretability algorithms helps understand the decision-making process and improve result interpretability. Future research will prioritize external validation of the existing predictive model and leverage longitudinal studies on colorectal cancer (CRC) to ascertain the model’s generalizability. We advocate for collaborative efforts among researchers to establish standardized, multicenter large-scale databases, thus augmenting the model’s generalizability and robustness, expediting its clinical application.

## 5 Conclusion

The LR model constructed in this study for predicting postoperative hospital stay duration in colorectal cancer patients demonstrated excellent predictive performance and interpretability, providing valuable information for healthcare efficiency evaluation and management. The analysis of feature variables’ impact on outcomes aids clinicians in understanding factors influencing patient hospital stay, providing a basis for healthcare professionals to implement personalized nursing interventions. This research offers support and guidance for clinical decision-making, potentially shortening patient hospital stays and reducing patients’ socio-economic burdens.

## Data availability statement

The raw data supporting the conclusions of this article will be made available by the authors, without undue reservation.

## Ethics statement

The studies involving humans were approved by Affiliated Hospital of Southwest Medical University (No.20190321-12). The studies were conducted in accordance with the local legislation and institutional requirements. The participants provided their written informed consent to participate in this study. Written informed consent was obtained from the individual(s) for the publication of any potentially identifiable images or data included in this article.

## Author contributions

ZW: Writing – review & editing, Writing – original draft. YW: Writing – original draft, Writing – review & editing. SC: Writing – review & editing, Writing – original draft. YL: Writing – review & editing, Writing – original draft. HD: Writing – original draft. HP: Writing – review & editing. SG: Writing – review & editing. PZ: Writing – review & editing. SZ: Writing – review & editing.
