# Supplementary material for: Construction of a predictive model for postoperative hospitalization time in colorectal cancer patients based on interpretable machine learning algorithm: a prospective preliminary study
Source: Front Oncol. 2024 Jun 14;14:1384931. doi: 10.3389/fonc.2024.1384931 (PMC11211394; doi:10.3389/fonc.2024.1384931)
Supplement: Supplementary file 1 [file DataSheet_1.docx]

**Supplementary Files：**

**Supplementary Files 1: Machine learning predictive model parameter**

RF (mtry=2, min_n=44, trees=248)

SVM (kernal=linear, C=0.2)

XGBoost (tree_depth=3, min_n=6)

LightGBM (tree_depth=1, min_n=7, trees=327)

DT (tree_depth=3, min_n=10)

KNN (neighbors=11)

CatBoost (iterations=100, depth=6, learning_rate=0.1, loss_function='Logloss', verbose=False)

DeepForest (n_estimators=100)

ANN (hidden_layer_sizes = (100,), max_iter=300)

**Supplementary Files 2: Baseline information analysis**

| Characteristics | hospitalization time≤8 days | hospitalization time＞8 days | P value |  |
| --- | --- | --- | --- | --- |
| n | 47 | 36 |  |  |
| Age, median (IQR) | 62 (53, 65.5) | 57.5 (50.5, 65) | 0.290 |  |
| Gender, n (%) |  |  | 0.174 |  |
| female | 24 (28.9%) | 13 (15.7%) |  |  |
| male | 23 (27.7%) | 23 (27.7%) |  |  |
| Height, mean ± sd | 1.5996 ± 0.073867 | 1.6192 ± 0.070968 | 0.227 |  |
| Weight, median (IQR) | 56 (49.25, 64.5) | 58 (53, 67.25) | 0.196 |  |
| BMI, median (IQR) | 22.032 (20.132, 23.581) | 22.268 (20.563, 26.477) | 0.314 |  |
| Smoking, n (%) |  |  | 0.006 |  |
| no | 41 (49.4%) | 22 (26.5%) |  |  |
| yes | 6 (7.2%) | 14 (16.9%) |  |  |
| Drinking, n (%) |  |  | 0.149 |  |
| no | 40 (48.2%) | 26 (31.3%) |  |  |
| yes | 7 (8.4%) | 10 (12%) |  |  |
| Occupation, n (%) |  |  | 0.520 |  |
| farmer | 36 (43.4%) | 24 (28.9%) |  |  |
| retiree | 4 (4.8%) | 5 (6%) |  |  |
| employee | 4 (4.8%) | 2 (2.4%) |  |  |
| worker | 3 (3.6%) | 5 (6%) |  |  |
| Medical Insurance, n (%) |  |  | 0.361 |  |
| yes | 47 (56.6%) | 34 (41%) |  |  |
| no | 0 (0%) | 2 (2.4%) |  |  |
| Underlying Conditions, n (%) |  |  | 0.643 |  |
| 1 kind | 17 (20.5%) | 14 (16.9%) |  |  |
| 0 kind | 25 (30.1%) | 16 (19.3%) |  |  |
| 2 kinds | 3 (3.6%) | 5 (6%) |  |  |
| 3 kinds | 2 (2.4%) | 1 (1.2%) |  |  |
| Tumor Location, n (%) |  |  | 0.838 |  |
| colon | 18 (21.7%) | 13 (15.7%) |  |  |
| rectum | 29 (34.9%) | 23 (27.7%) |  |  |
| Preoperative Hemoglobin, mean ± sd | 123.26 ± 19.288 | 126.53 ± 17.101 | 0.424 |  |
| Preoperative Albumin, mean ± sd | 41.738 ± 4.1414 | 42.394 ± 4.2133 | 0.480 |  |
| Total Protein, median (IQR) | 69.8 (65.45, 74.6) | 69.9 (64.075, 74.4) | 0.956 |  |
| Barthel, median (IQR) | 95 (90, 95) | 95 (90, 95) | 0.848 |  |
| KPS, n (%) |  |  | 0.697 |  |
| 90 | 33 (39.8%) | 23 (27.7%) |  |  |
| 80 | 12 (14.5%) | 10 (12%) |  |  |
| 100 | 2 (2.4%) | 3 (3.6%) |  |  |
| ZPS, n (%) |  |  | 0.415 |  |
| 0 | 18 (21.7%) | 17 (20.5%) |  |  |
| 1 | 29 (34.9%) | 19 (22.9%) |  |  |
| Stage, n (%) |  |  | 0.604 |  |
| 2 | 20 (24.1%) | 18 (21.7%) |  |  |
| 1 | 9 (10.8%) | 8 (9.6%) |  |  |
| 3 | 18 (21.7%) | 10 (12%) |  |  |
| Surgical Duration, mean ± sd | 219.79 ± 53.109 | 216.67 ± 52.003 | 0.790 |  |
| Blood Loss, median (IQR) | 100 (50, 175) | 50 (50, 100) | 0.004 |  |
| Pain Score, n (%) |  |  | 0.657 |  |
| 2 | 11 (13.3%) | 11 (13.3%) |  |  |
| 1 | 32 (38.6%) | 21 (25.3%) |  |  |
| 0 | 4 (4.8%) | 4 (4.8%) |  |  |
| QOR-15, median (IQR) | 134 (133, 136) | 135 (133, 136) | 0.706 |  |
| Postoperative frist-day movement distance, median (IQR) | 110 (89, 124.5) | 49.5 (0, 84.5) | < 0.001 |  |
| Steps count on the first day after surgery, median (IQR) | 234 (186.5, 259.5) | 105.5 (0, 183.75) | < 0.001 |  |
| Sleep duration on the first day after surgery, median (IQR) | 393 (325, 436.5) | 390 (352.25, 430.5) | 0.847 |  |
| Pain scores on the first day after surgery, median (IQR) | 4 (4, 5) | 5 (4, 5) | 0.172 |  |
| QOR-15 on the first day after surgery, mean ± sd | 79.787 ± 4.7178 | 77.556 ± 3.9384 | 0.025 |  |
| Postoperative second-day movement distance, median (IQR) | 275 (157, 334) | 100.5 (66.75, 198) | < 0.001 |  |
| Steps count on the second day after surgery, median (IQR) | 531 (329.5, 675.5) | 213.5 (138.5, 409.75) | < 0.001 |  |
| Sleep duration on the second day after surgery, median (IQR) | 410 (370, 438.5) | 400.5 (363.5, 427.75) | 0.629 |  |
| Pain scores on the second day after surgery, n (%) |  |  | 0.022 |  |
| 3 | 13 (15.7%) | 3 (3.6%) |  |  |
| 4 | 30 (36.1%) | 23 (27.7%) |  |  |
| 5 | 4 (4.8%) | 9 (10.8%) |  |  |
| 6 | 0 (0%) | 1 (1.2%) |  |  |
| QOR-15 on the second day after surgery, mean ± sd | 95.574 ± 4.6707 | 91.417 ± 4.2114 | < 0.001 |  |
| Postoperative third-day movement distance, median (IQR) | 398 (360, 458) | 287 (232.5, 322.25) | < 0.001 |  |
| Steps count on the third day after surgery, median (IQR) | 803 (711, 925.5) | 593.5 (474, 636.75) | < 0.001 |  |
| Sleep duration on the third day after surgery, median (IQR) | 419 (402, 447.5) | 405 (384.5, 440.5) | 0.180 |  |
| Pain scores on the third day after surgery, n (%) |  |  | < 0.001 |  |
| 2 | 15 (18.1%) | 2 (2.4%) |  |  |
| 3 | 25 (30.1%) | 14 (16.9%) |  |  |
| 4 | 7 (8.4%) | 20 (24.1%) |  |  |
| QOR-15 on the third day after surgery, mean ± sd | 105.66 ± 5.1257 | 100 ± 5.4195 | < 0.001 |  |
| Educational Level, n (%) |  |  | 0.150 |  |
| primary school | 26 (31.3%) | 14 (16.9%) |  |  |
| secondary school | 11 (13.3%) | 17 (20.5%) |  |  |
| university and above | 3 (3.6%) | 2 (2.4%) |  |  |
| illiterate | 7 (8.4%) | 3 (3.6%) |  |  |
| Complications, n (%) |  |  | 0.015 |  |
| no | 45 (54.2%) | 27 (32.5%) |  |  |
| yes | 2 (2.4%) | 9 (10.8%) |  |  |
|  |  |  |  |  |
